# From the Nurses' Point of View

**Published:** 1914-07-18

**Authors:** 


### FROM THE NURSES' POINT OF VIEW.

### A Reply to the Criticisms of a Patient.

[We have received the following article, which we have great pleasure in publishing, as a protest against the "Patient's Point of View" as expressed in a recent issue of The Hospital.]

The nurses of a private hospital in the West End feel it their duty to answer the article entitled "Hospitals from the Patients' Point of View" which appeared in The Hospital of June 20 last.

There are always two sides to every question, and we would like to offer some explanation and defence of what the paying patient considers "faults of administration" in private nursing institutions. However, primarily, we feel that the nurses (rather than the hospital) have been attacked; we suggest now giving the nurses' point of view with regard to the patients in nursing homes—and private hospitals in particular. The difference between the two is comparatively small, but for the benefit of an interested public, which perhaps knows very little of the inside working of these places, we give a brief explanation of both.

A nursing home as a rule is a large private house adapted more or less for nursing sick patients. Private rooms on each floor are generally used as rooms for the paying patient, and are charged according to size and position. The weekly nursing fees vary from £5 5s. to £20 (or more), and these fees do not include such extras as medicines, dressings, fires, etc. Usually there is a charge of one guinea made for the use of the operating theatre; the anæsthetist's fees vary from £2 2s. to £5 5s., and the operating surgeon's from £50 to £100. The nurses are all fully trained, and are made responsible for the care and treatment of two, or perhaps three, patients. They are on duty most of the day, and very rarely have even the comfort of a sitting room in which to have tea, or rest, in the intervals when the bells are not ringing.

A hospital for paying patients differs from a nursing home chiefly as regards the fees and the number of trained nurses employed. The place is generally built on hospital lines: several private rooms on each floor, bathrooms, lavatories, sink rooms, and kitchen; and the clearing of these places (excluding the kitchen) chiefly falls to the lot of the junior nurses or probationers, because such institutes are not as a rule endowed, and, therefore, cannot pay the wages of the number of maids necessary to do all the menial work. The staff might consist of a sister, trained nurse, assistant (partially trained), and probationer, to attend to ten or twelve private patients in separate rooms. The nurses' off-duty times are arranged much the same as in a general hospital, and are taken during the day's work at stated times. Also half an hour is allowed for each nurse for each meal. This arrangement of nurses naturally lowers the expenses considerably; the sister and staff nurses receive salaries varying from £28 to £35, while the assistant may be paid at the rate of £24, the probationer receiving no salary at all.

The patient who pays two or three guineas a week for treatment, surgical operations, food, room, nursing, and medical attendance, inclusive, is undoubtedly receiving charity, and has no right to expect the same trained attention as he might demand at an expensive nursing home.

The patients in all nursing homes and private hospitals are dependent on bells to summon the nurses, and we can honestly say that in the greater number of cases this means of communication is shamefully abused. Individual patients are only too apt to forget that, besides themselves, there are many other patients in the same building who are entitled to exactly the same amount of care and attention. It is usually the convalescent patient (or those who have had a comparatively small operation), who ring the bell for trivial things, or to ask the nurse to have a chocolate from a box which has just arrived; thus calling the nurse from more serious cases requiring her attention. We would also like to remind the convalescent patient that in a hospital for surgical operations the beds are being refilled with fresh cases almost daily, necessitating fresh operations, and many of a very serious nature. Certainly the critical cases demand the first attention (and possibly no one but a nurse who has had actual experience can realise how much time must be devoted to make one such case even reasonably comfortable). It is at such times that delay is so often caused in answering the bells of convalescent patients who really have no immediate need but have acquired a wish for something. It is not unusual for a patient to ring and ask the nurse, not the maid, to replenish the fire or close the window, although there are several visitors in the room. Or, again, a patient has been known, after discussing the nurses with a visitor, to ring the bell violently many times in succession, without giving the nurse reasonable time to reach the room, to "show" the nurse whom she fancies most!

If patients would be more charitable in their criticism and treat the nurses who attend them as gentlewomen instead of drudges, we have no doubt the compliment would be returned. Natural resentment on the part of the nurses is often the cause of delay in attending to the patients who treat them as menials.

Oh, God, that men could see a little clearer,
Or judge less hardly where they cannot see!

In conclusion, we would like to suggest that the patient in bed is generally in an abnormal state of health, and is apt to become hypercritical; on the other hand a nurse's life is a constant nerve strain, and wearing both to body and mind, and, as Disraeli so aptly says, "It is far more easy to be critical than correct."

